# Comparative genomics of an endophytic *Pseudomonas putida* isolated from mango orchard

**DOI:** 10.1590/1678-4685-GMB-2015-0186

**Published:** 2016-07-07

**Authors:** Huma Asif, David J. Studholme, Asifullah Khan, M. Aurongzeb, Ishtiaq A. Khan, M. Kamran Azim

**Affiliations:** 1 Jamil-ur-Rahman Center for Genome Research, Dr. Panjwani Center for Molecular Medicine and Drug Research, International Center for Chemical and Biological Sciences, University of Karachi, Karachi, Pakistan; 2 Biosciences, University of Exeter, Exeter, U.K.

**Keywords:** Mangifera indica, bacterial genomics, plant-associated bacteria, endophyte

## Abstract

We analyzed the genome sequence of an endophytic bacterial strain *Pseudomonas putida* TJI51 isolated from mango bark tissues. Next generation DNA sequencing and short read de novo assembly generated the 5,805,096 bp draft genome of *P. putida* TJI51. Out of 6,036 protein coding genes in *P. putida* TJI51 sequences, 4,367 (72%) were annotated with functional specifications, while the remaining encoded hypothetical proteins. Comparative genome sequence analysis revealed that the *P. putida* TJI51genome contains several regions, not identified in so far sequenced *P. putida* genomes. Some of these regions were predicted to encode enzymes, including acetylornithine deacetylase, betaine aldehyde dehydrogenase, aldehyde dehydrogenase, benzoylformate decarboxylase, hydroxyacylglutathione hydrolase, and uroporphyrinogen decarboxylase. The genome of *P. putida* TJI51 contained three nonribosomal peptide synthetase gene clusters. Genome sequence analysis of *P. putida*TJI51 identified this bacterium as an endophytic resident. The endophytic fitness might be linked with alginate, which facilitates bacterial colonization in plant tissues. Genome sequence analysis shed light on the presence of a diverse spectrum of metabolic activities and adaptation of this isolate to various niches.

## Introduction

The genus *Pseudomonas* is a versatile and ecologically important group of bacteria. They are Gram-negative, slightly curved flagellated rods and prolific colonizers of surfaces (Clarke, 1982). *Pseudomonas* species have been isolated from diverse ecosystems including marine, freshwater and terrestrial environment including plants and animals sources (Achouak *et al.*, 2000; Manaia and Moore, 2002; Liu *et al.*, 2008). This widespread distribution is due to physiological and genetic diversity (Spiers *et al.*, 2000). For instance, an attempt to differentiate *P. Stutzeri* populations from a variety of ecological niches resulted in several distinguishable genomovars (Rossello *et al.*, 1991). Comparative genomics of the *Pseudomonas* strains revealed much variability in their genome sizes, ranging from 3.7 Mbp for *Pseudomonas stutzeri* to 7.1 Mbp for *Pseudomonas aeruginosa* (Schmidt *et al.,* 1996; Ginard *et al.*, 1997).

*Pseudomonas putida,* an important member of genus *Pseudomonas,* is frequently found in temperate waters and diverse soil environments. It is renowned for its ability to degrade a wide variety of natural and man-made compounds, and thus plays an important role in maintaining environmental quality (Dejonghe *et al.*, 2001). Available complete or draft genome sequences of several *P. putida* isolates from different parts of the world provided a rich and diverse 'meta-dataset' (Genome OnLine Database; www.genomeonline.org). *Pseudomonas putida* KT2440 isolated in Japan is so far, the best characterized strain, which is a plasmid free derivative of *Pseudomonas putida*mt-2 (Timmis, 2002). Other strains with sequenced genomes include *Pseudomonas putida* W619, a plant growth-promoting endophytic bacterium, *Pseudomonas putida* F1, the aromatic hydrocarbon degrading bacterium, and *Pseudomonas putida* GB-1, a robust manganese (Mn^2+^) oxidizer. *Pseudomonas putida* W619 and *Pseudomonas putida* KT2440 have been found in association with plants (Wu *et al.*, 2011). However, genome sequences representing more *P. putida* strains are required to better determine the prevailing diversity and stratification patterns of this environmentally important bacterium. Here, we report the draft genome sequence of *Pseudomonas putida* TJI51 isolated from infected mango bark. The comparative sequence analysis revealed several genomic loci specific to this endophytic *Pseudomonas putida* isolate.

## Material and Methods

### Isolation and bacteriological characterization of *Pseudomonas putida* TJI51

Isolation of *Pseudomonas putida* TJI51 has been described elsewhere (Khan *et al.*, 2014). Briefly, *Pseudomonas putida* TJI51 was isolated from bark of a mango tree situated in the Horticultural Garden, Sindh Agriculture University, Tando Jam, Pakistan. The bark sample (100 mg) was surface sterilized using 1.3% sodium hypochlorite solution, followed by homogenization using sea sand and 0.8% NaCl. The homogenate was incubated on LB plates and isolated colonies were characterized further. Colony PCR of isolated bacterial colonies was performed according to Khan *et al.* (2014) for amplification of 16S ribosomal DNA. Sanger sequencing of PCR products was carried out, followed by BLASTN analysis (Altschul*et al.,* 1990) of resulted sequences against the NCBI 16S ribosomal RNA sequence (Bacteria and Archaea) database.

Bacteriological analysis was done using a standard protocol for *Pseudomonas* identification, including sulphur, indole, citrate, motility, urease, and TSI agar tests. Tests for lactose and glucose fermentation, PSP (Pseudomonas agar Pyocyanin) and PSF (Pseudomonas agar Flourescein) were also carried out (Murray *et al.*, 2007). Antibiotic susceptibility tests were carried out using disc diffusion method with the following antibiotics discs (Oxoid Ltd. England): Ceftriaxone (30 μg), Cefixime (5 mg) and Ceftazidime (30 μg) (3rd-generation cephalosporins); Cefuroxime (30 μg) (2nd generation cephalosporin); Ciprofloxacin (5 μg) (Quinolone); Sulfamethoxazole (25 μg) (Sulfonamide); Polymyxin B (300 units) (Polypeptides); Meropenem (10 μg), Imipenem (10 μg) (Carbapenems); Tetracycline (30 μg) (Tetracyclines); Amoxicillin/clavulanate (30 μg), Tazobactam (110 μg) (Penicillin combinations); Gentamicin (10 μg), Amikacin (30 μg) (Aminoglycosides); Aztreonam (30 μg) (Monobactams) and Chloramphenicol (30 μg). Bacterial isolates were also inoculated on casein agar and blood agar (Blood agar base, Oxoid code CM 271) for determination of proteolytic and haemolytic activities.

### Genome sequencing and data analysis

Genomic DNA (5 μg) was purified from an overnight culture of *P. putida* TJI51 using a bacterial genomic DNA isolation kit (Bio Basic Inc.) and subjected to Illumina next generation sequencing. A paired-end library of insert size 500 bp was prepared according to the manufacturer's protocol followed by HiSeq2000 system sequencing (Illumina Inc., San Diego, USA). The obtained raw sequence data was subjected to filtering of low quality score reads (i.e. < Q20) using the FastX toolkit (http://hannonlab.cshl.edu/fastx_toolkit/). CLC Genomics Workbench version 7.5.2 was used for denovo paired-end sequence assembly. The annotation of the assembled genome sequences was carried out using the NCBI Prokaryotic Genomes Automatic Annotation Pipeline (PGAAP) (http://www.ncbi.nlm.nih.gov/genomes/static/Pipeline.html).

### Comparative genome analysis

We used MUMMER (Delcher *et al.*, 1999) to align assembled contigs with several *Pseudomonas* genome sequences. A molecular phylogeny analysis of *P. putida* TJ151 was carried out with several housekeeping gene sequences and multi-locus sequence analysis (MLSA) using MEGA version 4.0 (neighbor-joining method) (Saitou and Nei, 1987). The comparative analyses with available bacterial genome sequences were performed using BLAST programs (BLASTN, TBLASTX and BLASTP) (Altschul *et al.,* 1990). The comparative genomics analysis of *P. putida* TJ151 was carried out with *Pseudomonas putida* KT2440 (NC_002947), *Pseudomonas putida* GB-1 (NC_010322), *Pseudomonas putida* w619 (NC_010501), *Pseudomonas putida* F1 (NC_009512), *Pseudomonas fluorescens* Pf5 (NC_004129), *Pseudomonas syringae* pv. *Tomato* DC3000 (NC_004578), *Pseudomonas syringae* pv. *Phaseolicola* 1448A (NC_005773), *Pseudomonas entomophila* L48 (NC_008027), *Pseudomonas aeruginosa* UCBPP-PA14 (NC_008463), *Pseudomonas aeruginosa* PA7 (NC_009656), *Pseudomonas aeruginosa LESB58* (NC_011770), *Pseudomonas fluorescens* SBW25 (NC_012660) and *Pseudomonas fluorescens Pf01* (NC_007492) using SEED viewer (Dejongh *et al.*, 2007). ClustalX version 2.0 (Larkin *et al.*, 2007) and MEGA4 software (Tamura *et al.*, 2007) were used for multiple sequence alignment and phylogenetic tree construction respectively.

A comparison of genomic DNA G+C content between *P. putida* TJ151 and other *Pseudomonas* strains was done using the Integrated Microbial Genomes (IMG) system (Markowitz *et al.*, 2010). The sequence analysis of biosynthetic gene clusters for bacterial secondary metabolites was carried out by antiSMASH 2.0 web server (Blin *et al.*, 2013). The association of metabolic pathways with the annotated protein sequences was studied using the KEGG server (Kanehisa *et al.*, 2008).

## Results

We isolated a number of endophytic *Pseudomonas* strains from dead tissues of bark, leaves and inflorescence of mango (*Mangifera indica*) trees grown in agricultural farms in mango growing districts in Sindh province of Pakistan (Khan *et al.*, 2014). The bacterial characterization was carried out by standard procedures and 16S ribosomal DNA sequencing, which identified isolate-number TJI51 as *Pseudomonas* species. Antibiotics susceptibility assays of *Pseudomonas* sp.TJI51 showed resistance against Ampicilin, Cefixime (3^rd^ generation cephalosporin) and Sulfamethoxazole (Sulfonamide). *Pseudomonas* sp. TJI51 was found to be oxidase and citrate positive; nonhemolytic, nonproteolytic, a non-lactose fermenter and motile. Genome-wide DNA sequencing and comparative genomics were done for detailed functional characterization of this isolate.

### Genome sequence of *Pseudomonas putida* TJI51

The sequencing of chromosomal and plasmid DNA of *Pseudomonas* sp. TJI51 resulted in 651 mega bases (Mb) of raw data with read length of 90 bp. During raw data filtering by the FASTX-Toolkit, low quality bases (≤ Q20 bases) at 3 ends of the sequence reads, adaptor sequences and reads containing Ns (undetermined/ambiguous nucleotides) were removed. Accordingly, 5,777,778 reads of 90 bp length were obtained (total nucleotides=520,000,020). De novo assembly of sequence reads using CLC Genomics Workbench resulted in 208 contigs of ≥ 541 bp length. Total sequence length of the assembly was 5,805,096 bp, which was in accordance with the previously reported 6.1 Mb genome of *P. Putida* (Nelson *et al.*, 2002; Moon *et al.*, 2008; Peix*et al.,* 2009). The N_50_ of assembly was 53,388 bp and the length of the longest contig was 240,918bp. The depth of coverage was estimated as 89X, and mean G+C content was 62% (Table 1). The G+C content distribution plot showed that most of the scaffolds had the GC content in the range of 58-64%, which is in agreement with other *Pseudomonas* strains. The master record for the genome shotgun sequencing project of *P.* species TJI51 can be accessed under GenBank under accession number AEWE00000000.2.

**Table 1:** Sequencing and assembly data of *P. putida TJ151* genome.

Library characteristics	Data
No. of filtered paired-end reads	5,777,778
Read length	90
No. of filtered nucleotide bases	520,000,020
Number of assembled contigs	208
Length of longest contigs (nucleotides)	240,918
Total length of contigs (nucleotides)	5,805,096
N50 of contigs (nucleotides)	53,388
Fold coverage	89 X

### Comparative genomics of *Pseudomonas* sp. TJ151

**Figure 1:**
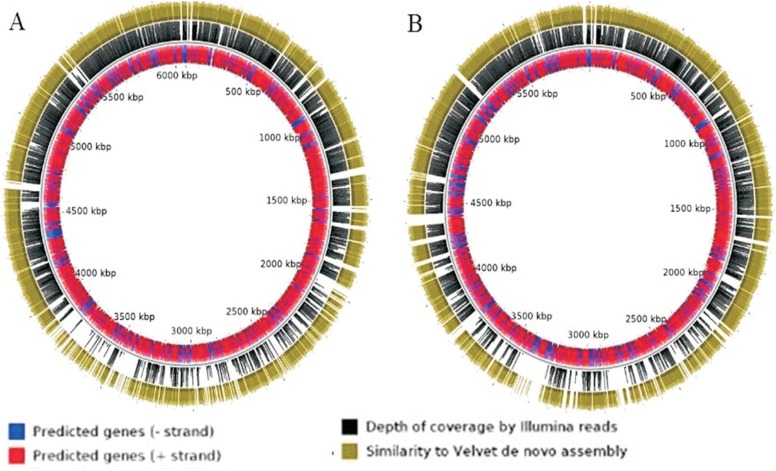
Diagrammatic representation of MUMMER (Delcher *et al.,* 1999) generated genome-wide alignments of *P. putida* TJI51 contigs with genome sequences of *P. putida* GB-1 (A) and *P. putida* F1 (B).

The 208 scaffolds of the *Pseudomonas* sp. TJ151 genome sequence were aligned with 13 *Pseudomonas* genomic sequences using MUMMER (Delcher *et al.*, 1999). The analysis showed that *P. putida* sequences aligned with greater proportion to *P. sp.* TJI51 sequences (Table 2). The highest percentages of aligned nucleotides were for *P. putida* GB-1, F1 and KT2440 (Figure 1). The genome-wide alignment showed that 33-35% of the *P.* sp. TJ1-51 sequences were aligned with *P. putida* GB-1, *P. putida* F1, and *P. putida* KT2440; 21% with *Pseudomonas putida* W619 genome; 19% with *P. entomophila,* while 2-4.5% with *Pseudomonas syringae, P. fluorescens,* and *P. aeruginosa* genomic sequences. Hence, these alignments identified *P.* sp. TJI51 as being more similar to *P. Putida* than to the other species included in this analysis.

**Table 2:** Genome-wide pair-wise sequence alignment of *P. putida* TJ151 with 13 *Pseudomonas* species.

*Pseudomonas* genomes	GenBank accession number	Genome size	*Pseudomonas putida* TJI51 nucleotides aligned	Genome coverage (%)	GC content (%)
*Pseudomonas putida* KT2440	NC_002947	6181861	2068300	33.457	62
*Pseudomonas putida* GB-1	NC_010322	6078430	2152707	35.415	62
*Pseudomonas putida F1*	NC_009512	5959964	1987103	33.340	62
*Pseudomonas putida* w619	NC_010501	5774330	1259208	21.806	61
*Pseudomonas entomophila*L48	NC_008027	5888632	1121068	19.037	64
*Pseudomonas fluorescens*Pf5	NC_004129	7072643	311819	4.408	61
*Pseudomonassyringaepv. tomatostr*. DC3000	NC_004578	6386667	117603	1.841	58
*Pseudomonassyringaepv. phaseolicola*1448A	NC_005773	5917006	113979	1.926	58
*Pseudomonas aeruginosa* UCBPP-PA14	NC_008463	6523118	144831	2.220	66
*Pseudomonas aeruginosa* PA7	NC_009656	6576011	163735	2.489	66
*Pseudomonas aeruginosa* LESB58	NC_011770	6054647	18562	0.306	66
*Pseudomonas fluorescens*SBW25	NC_012660	6720050	216725	3.225	60
*Pseudomonas fluorescens*Pf01	NC_007492	6436448	256766	3.989	61

**Figure 2:**
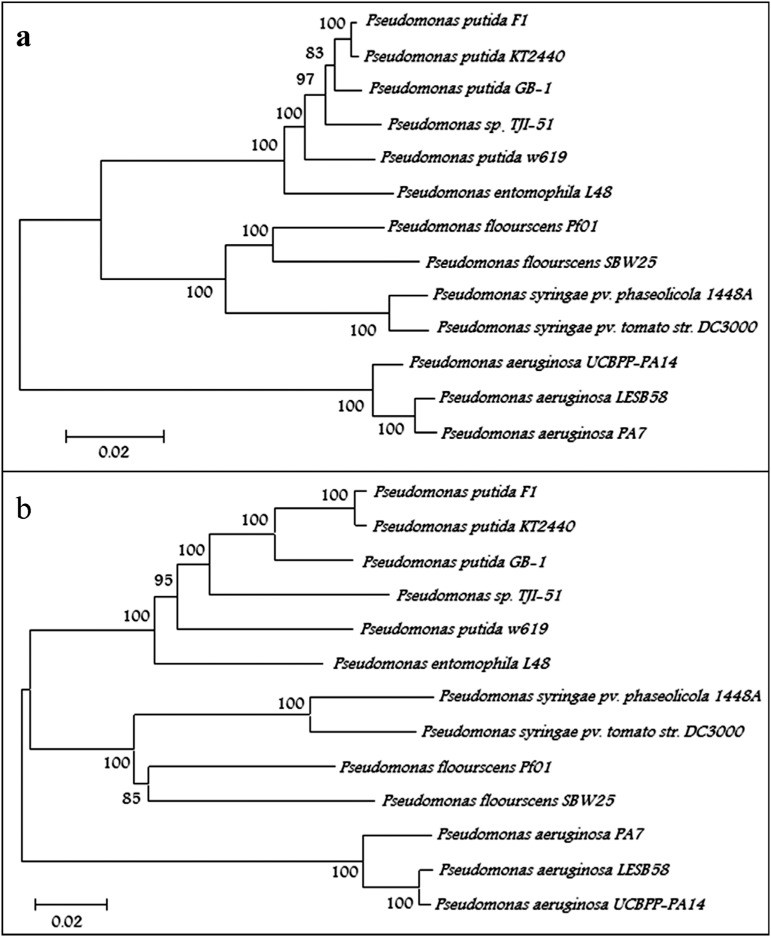
Neighbor-Joining phylogenetic trees of *P. putida* TJI51 and other *Pseudomonas* species based on (A) concatenated sequence of five housekeeping genes (*gyrB, lepA, recA, recG* and *rpoD)* and (B) concatenated amino acid sequence of ten conserved protein sequences. Bootstrap values are represented on the horizontal branches of the trees.

Further support regarding the classification of *P.* sp. TJI51 as *P. Putida* was obtained from phylogenetic studies. DNA sequences of five conserved housekeeping genes, *gyrB, lepA, recA, recG* and *rpoD,* were selected for the phylogenetic analysis of *P.* sp. TJ151 (Santos and Ochman, 2004). The corresponding gene sequences of 13 *Pseudomonas* strains selected on the basis of BLAST results were retrieved from GenBank (Table 2) (Madden *et al.*, 1996). In the resultant phylogenetic tree, *P.* sp. TJ151 grouped with *P. putida* GB-1 and *P. putida* w619, with boot strap support values of 97 and 100% respectively (Figure 2A). Moreover, a phylogenetic analysis based on the concatenated amino acid sequence data of ten orthologous conserved proteins was also carried out. These universally distributed bacterial genes included transfer RNA synthetases, translation elongation factor, and DNA-directed RNA polymerase beta subunit (Brown *et al.*, 2001). Amino acid sequence alignment of these genes was made using the NJ method. This analysis also showed the alliance of *P. sp. TJ151* with *P. putida* GB-1 and *P. putida* w619, with bootstrap support values 97 and 100% respectively (Figure 2B). The graphical representation of genome-wide sequence alignment indicated that *P. putida* TJ151 sequences covered most of the genome map of *P. putida* GB-1 and *P. putida* F1 with several gap regions (Figure 1).

### Annotation of *Pseudomonas putida* TJ151 genome

**Figure 3:**
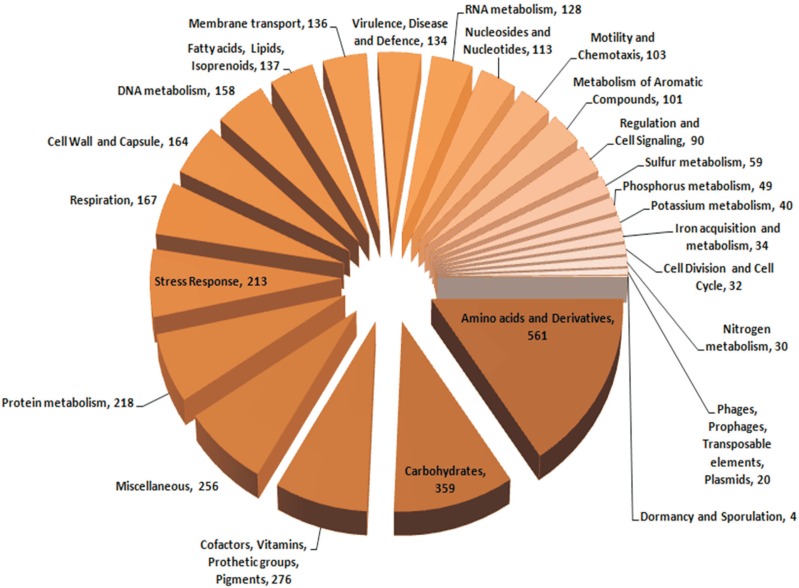
Annotation of *P. putida* TJ151 genes by the NCBI Prokaryotic Genomes Automatic Annotation Pipeline (PGAAP) and their distribution according to different biochemical categories.

The NCBI Prokaryotic Genome Annotation Pipeline (PGAAP) annotated 6,036 protein coding genes in *P. putida* TJI51 sequences. In total, 72% of protein coding genes (4,367) were annotated with functional specifications, while the remaining werehypothetical proteins (1,669). The distribution of these genes in different bacterial subsystems is shown in Figure 3. Gene ontology by PGAAP identified protein coding genes involved in core and accessory functions. Genes involved in core functions included nucleic acid biosynthesis, amino acid metabolism, carbohydrate metabolism, protein metabolism, cell wall and capsule synthesis, respiration, etc. A significant number of genes encoding accessory functions were also found, including iron acquisition, metabolism of aromatic compounds, stress response motility and chemotaxis.

## Discussion

We characterized the genome of an endophytic *P. putida* strain isolated from mango tree located in an agricultural farm in Sindh province of Pakistan. Genome insight provided information regarding lifestyle and adaptation of *P. putida* TJI51. Plant-associated *Pseudomonas* species have rarely been reported from this part of the world. The genetic repository of *P. putida* TJI51 allowed comparative genomics and functional analysis with available *Pseudomonas* genomes for information related to the mode of association with plant tissues.

A sizeable number of genes were predicted to encode hypothetical proteins (i.e. 28%). Analyses of newly sequenced bacterial genomes constantly reveal novelprotein coding genes; hence the size of the 'pan-genome' is increasing (Tettelin *et al.,* 2008). BLAST searches in the NCBI non-redundant protein sequence database revealed 37 novel hypothetical proteins in *P. putida* TJI51 that have no database match (11 of these novel hypothetical proteins had ≥ 50 amino acids, including hypothetical protein G1E_25761 which contained 1295 amino acids).

Analysis of the genome-wide alignment revealed 5-15 kb regions in *P. putida* TJ151 genome sequences that shared no detectable nucleotide sequence similarity with previously sequenced *P. putida* genomes. These regions were predicted to encode proteins involved in an array of biochemical pathways, including different oxidoreductases, glutathione S-transferase, isopenicillin N-synthase, xanthine permease, acetylornithine deacetylase, aldehyde dehydrogenase, benzoylformate decarboxylase, endoribonuclease, different 2Fe-2S proteins, ABC transporters, extracellular solute-binding protein, xanthine dehydrogenase, hydroxyacylglutathione hydrolase, glucose dehydrogenase, uroporphyrinogen decarboxylase, and N-acetylglucosamine-binding protein. The *P. putida* TJ151 genome also appears to contain two plasmids (i.e. contig 20; AEWE02000020 and contig 77; AEWE02000077) and phage sequences (AEWE02000074). Contig 20 aligned with plasmid pGRT1 of the *P. putida* strain DOT-T1E, whereas Contig 77 aligned with plasmid sequences of several *P. putida* strains, including GB-1 and W619. Also among the *P. putida* TJ51-specific regions were genes predicted to encode resistance to heavy metals (contigs represented by GenBank entries AEWE02000041 and AEWE02000128), which may represent adaptations to treatment of the plant host environment with antimicrobial copper sulphate. The study generally augments the knowledge of the pan-genome of the ubiquitous and metabolically versatile species *P. putida*.

### ABC transporters in *Pseudomonas putida* TJI51

The ATP-binding cassette (ABC) transporters family of proteins are common in archaea, bacteria and eukaryotes. They facilitate active transport of an array of substrates, including ions, sugars, lipids, sterols, peptides, proteins, and drugs (Higgins, 1992). Bacterial ABC transporters typically are composed of three components; two integral membrane proteins, each having six transmembrane segments, two peripheral ATPase subunits, and a periplasmic substrate-binding protein. Usually, the genes for the three components form operons, as observed in many prokaryotic genomes.

KEGG analysis revealed 137 ABC transporter system genes in the *P. putida* TJI51 genome sequence. We found a complete set of ATP transporter genes (i.e. integral membrane protein, ATPase and substrate-binding protein) for glycine/betaine/L-proline, different canonical amino acids, phosphate, sulfate, choline, urea, taurine, lipid, metal (i.e. Fe+3, molybdate, nickel), oligopeptide/dipeptide, microcin C, and spermidine/putrescine. Putrescine, or tetramethylenediamine (the precursor of spermidine), is a foul-smelling compound produced by the amino acid catabolism in living and dead organisms. Our analysis showed four distinct orthologous ABC transporter system gene sets for spermidine/putrescine in *P. Putida* TJI51. Hence, it appeared that *P. putida* TJI51 contains redundant sets of spermidine/putrescine ABC transporters. This observation is consistent with the fact that the isolate was obtained from dead mango bark tissues. We noted that the draft genome sequence of *P. putida* TJI51 did not contain complete operon sequences of these ATP transporter systems. More than one third of the 137 ATP transporter genes, i.e. 49 (36%), were annotated as ATP transporter protein or ATP transporter-like protein. Hence, their substrate information could not be ascertained.

In *P. putida*TJI51, the *liv* gene cluster *LIV*-I, specific for the branched-chain amino acid transport system, was comprised of a periplasmic binding protein LivK, two permease domains LivH and LivM, and two ATP-binding subunits LivF and LivG. Closely related orthologues of the *liv* gene cluster are found in other *Pseudomonas* species. This cluster is found functional in *P. putida* TJI51 and predicted to be involved in amino acid transport.

### TypeVI secretion system (T6SS) in *Pseudomonas putida* TJ151

The type VI secretion system (T6SS) gene cluster is widespread among non-pathogenic and pathogenic gram negative bacteria (Boyer *et al.*, 2009). This system enables the bacterial species to maintain pathogenic or symbiotic interaction with their eukaryotic host. The secretion systems facilitate the extracellular transport of proteins into the target eukaryotic cells without requiring hydrophobic amino-terminal sequences (Pukatzki *et al.*, 2006). The T6SS gene cluster comprises 14 core genes that vary in composition between different bacterial species (Bingle *et al.*, 2008). Virulence associated secretion (*vas*) genes secreted by the T6SS are shown to be responsible *for Vibrio cholera* cytotoxicity towards *Dictyostelium amoebae* and mammalian J774 macrophages (Pukatzki *et al.*, 2006). A survey showed that T6SS is present mostly in the non-pathogenic bacteria or symbionts, e.g. *Myxococcus xanthus* and *P. putida* (Bingle *et al.*, 2008). However, more recently it has been reported to be involved in the virulence of *Burkholderia mallei* (Schell *et al.*, 2007).

**Figure 4:**
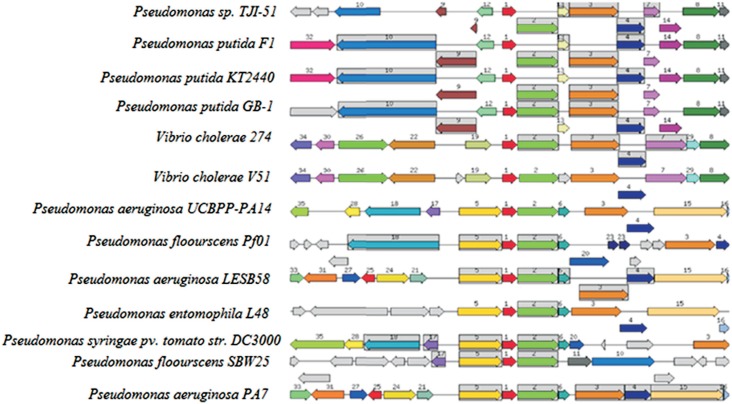
Gene organization of Type VI secretion clusters. Homologous genes are grouped and shown with same number and color. Functionally coupled genes are highlighted in gray background boxes (*ImpG/VasA* (numbered 3, orange arrow), ImpC protein (numbered 2, green arrow), *ImpH/VasB* (numbered 4, blue arrow) and *ImpI/VasC* (numbered 7, purple arrow).

Bioinformatics analyses identified *Vas* genes to be highly conserved in Gram-negative bacteria. The comparison of the T6SS gene cluster in *Pseudomonas putida* TJI51 with other bacterial strains showed a closely related gene organization (Figure 4). The genes ImpG/VasA (numbered 3), Uncharacterized protein ImpC (numbered 2) and ImpH/VasB (numbered 4) were present at conserved relative positions, while the ImpI/VasC gene (numbered 7) was conserved in few strains.

### Association of *Pseudomonas putida* TJ151 with plants

*P. putida* TJI51 was found to live in association with mango tree bark as an endophyte, and comparative genomics was used to identify the possible functions that are involved in the association of this bacterium with its host.

KEGG analysis of the *P. putida* TJI51 genome revealed 26 genes involved in flagellar biosynthesis. All genes required for flagellar assembly were found present, including the *flhB* gene. It was reported that the impaired swimming capability of *P. putida* DOT-T1E (Segura *et al.*, 2004) resulted from a mutation in the *flhB* gene. The *P. putida* TJI51 *flhB* gene is 91% identical to that of *P. Putida* KT2440, which is known to be a good swimmer (Wu *et al.*, 2011). These observations indicated a probable swimming capability of *P. putida* TJI51. Moreover, the KEGG analysis of the *P. putida TJI51* genome revealed genes for all proteins involved in bacterial chemotaxis. Multiple genes for CheA (n=7) and CheW (n=10) histidine kinases were found. Moreover, *P. putida TJI51* also contains RbsB and DppA encoding genes involved in D-ribose and dipeptide chemotaxis in *E. coli*.

### Metabolism of secondary metabolites by *Pseudomonas putida* TJ151

Many bacteria synthesize natural products with significant bioactivities, including antibiotics, anticancer agents, and other chemotherapeutics (Newman and Cragg, 2012). Microbial genome mining is a promising alternative for labor-intensive and time-consuming methods to identify and characterize bioactive secondary metabolites. The genome sequence analysis of *P. putida* TJI51 carried out by antiSMASH (Blin *et al.*, 2013) and KEGG servers pointed out several genes putatively involved in the synthesis and catabolism of such metabolites. The antiSMASH (ANTIbiotics and Secondary Metabolites Analysis SHell) combines the automated identification of secondary metabolite gene clusters in genome sequences with a large collection of compound-specific analysis algorithms (Blin *et al.*, 2013). AntiSMASH identified at least three non-ribosomal peptide synthetase gene clusters in *P. putida* TJI51 sequences. Non-ribosomal peptides produced by bacteria and fungi are a diverse family of secondary metabolites with a broad range of bioactivities (Schwarzer*et al.,* 2003). Nonribosomal peptides are antibiotics, cytostatics and immunosuppressants, siderophores, or pigments. These peptides are synthesized by non-ribosomal peptide synthetases (NRPS) (Strieker *et al.,* 2010). In bacteria, the NRPS genes for a certain peptide are usually organized in one operon in bacteria. The NRPS are organized in modules, where each module consists of several domains with defined functions. The domains of a complete NRPS include an adenylation domain (A-domain), thiolation and peptide carrier protein with attached phosphopantetheine (PCP domain), condensation domain for amide bond formation (C- domain), thioesterase domain for termination (TE domain), and an optional epimerization into D-amino acids domain (E-domain).

Two NRPS gene clusters found in *P. Putida* TJI51sequences were located in the following contigs. (1) Contig 131 (GenBank accession AEWE02000131) contained two sets of A-domain, PCP-domain and condensation domain. NRPS gene sequences in this contig were homologous to *Burkholderia pseudomallei 1710b*. (2) Contig82 (GenBank accession AEWE02000082) contained a novel set of A-domain, PCP-domain, condensation domain, along with an epimerization domain.

The genome analysis of *P. putida* TJ151by the KEGG server revealed catabolic pathways for the transformation of bioactive aromatic compounds, including L-tyrosine, Azathioprine/6-Mercaptoprine and Fluorouracil. Azathioprine is an immunosuppressive prodrug which is almost completely converted to 6-Mercaptoprine (Maltzman and Koretzky, 2003), whereas Fluorouracil is an anticancer drug. The KEGG analysis identified nine enzymes involved in biodegradation of these xenobiotics in the *P. putida* TJI51 genome.
